# Free Radical and Viral Infection: A Review from the Perspective of Ferroptosis

**DOI:** 10.3390/vetsci10070456

**Published:** 2023-07-11

**Authors:** Jun Chen, Jinping Fu, Sha Zhao, Xiaoxi Zhang, Yuyang Chao, Qunxing Pan, Huawei Sun, Jingfeng Zhang, Bin Li, Tao Xue, Jingui Li, Chuanmin Liu

**Affiliations:** 1 College of Veterinary Medicine, Yangzhou University, Yangzhou 225009, China; junchenjs@126.com; 2 Institute of Veterinary Medicine, Jiangsu Academy of Agricultural Sciences, Nanjing 210014, China; jinping@ksu.edu (J.F.);; 3 Key Laboratory of Veterinary Biological Engineering and Technology, Ministry of Agriculture, Nanjing 210014, China; 4 Key Laboratory of Veterinary Diagnosis, Jiangsu Academy of Agricultural Sciences, Nanjing 210014, China; 5 College of Medicine, Linyi University, Linyi 276000, China; 6 School of Life Sciences, Jiangsu University, Zhenjiang 212013, China; 7 College of Veterinary Medicine, Nanjing Agricultural University, Nanjing 210095, China

**Keywords:** viral infection, free radical, mitochondrion, ferroptosis

## Abstract

**Simple Summary:**

Ferroptosis is an iron-catalyzed, oxidative form of cell death. It is accompanied by a large amount of iron accumulation and lipid peroxidation. Increasingly, studies have demonstrated that ferroptosis is closely associated with various organ injuries and degenerative pathologies, among others. This review aims to investigate the molecular mechanism between free radicals and ferroptosis in the viral infection state. This review also discusses the possibility of using ferroptosis inducers and inhibitors to treat viral diseases, providing some theoretical basis for studying the pathogenesis and prevention of viruses.

**Abstract:**

Free radicals, including reactive oxygen species (ROS) and reactive nitrogen species (RNS), play critical roles in various physiological activities such as cell differentiation, apoptosis, and vascular tension when existing in cells at low levels. However, excessive amounts of free radicals are harmful, causing DNA damage, lipid peroxidation, protein degeneration, and abnormal cell death. Certain viral infections induce cells to produce excessive free radicals, which in multiple ways help the virus to replicate, mature, and exit. Iron is a necessary element for many intracellular enzymes, involved in both cellular activities and viral replication. Ferroptosis, a programmed cell death mode distinct from apoptosis, necrosis, and pyroptosis, is characterized by lipid peroxide accumulation and damage to the antioxidant system, affecting many cellular processes. Viral infection commonly manifests as decreased glutathione (GSH) content and down-regulated glutathione peroxidase 4 (GPX4) activity, similar to ferroptosis. Recent studies have suggested a possible relationship among free radicals, viral infections and ferroptosis. This review aims to elucidate the molecular mechanism linking free radicals and ferroptosis during viral infections and provide a new theoretical basis for studying viral pathogenesis and control.

## 1. Introduction

Viruses are ubiquitous in nature and the majority of them have pathogenic effects. In modern life, large populations as well as rapid transportation, have increased the risk of pandemics and epidemics, leading to significant social and economic losses. In recent years, African swine fever virus, which can cause acute haemorrhagic fever, has been spreading from Africa to Europe [1]. In 2018, the virus successfully infiltrated China and other countries in Southeast Asia, posing a threat to global pork production and safety. Additionally, in 2019, the coronavirus disease 2019 (COVID-19), caused by severe acute respiratory syndrome coronavirus 2 (SARS-CoV-2), rapidly spread across the globe [2]. The COVID-19 pandemic has had an unpredictable impact on various aspects of society, including healthcare facilities, food security, global economic depression, psychosocial conditions, quality of life, and so on [3,4,5].

For replication purposes, the virus exploits the resources of host cells. In response, host cells rapidly activate their immune system to halt virus replication, eliminate the virus, and repair damaged areas. However, in the process of immune activation, host cells generate an excess of free radicals [6]. Normally, free radicals maintain a balance with the antioxidant system and play important roles in regulating vascular tone, promoting immunity, acting as signaling molecules, and regulating cell aging. Nevertheless, viruses induce the body to produce an excessive amount of free radicals, leading to redox imbalance and oxidative stress [7]. The relationship between free radicals and virus infection is complex. On the one hand, viral stimulation of cells to produce excess free radicals can trigger cell death [8]. However, cell death is an intrinsic immune defense mechanism in which the host sacrifices infected cells to preserve the remaining normal cells and keep the host alive [9,10]. On the other hand, some free radicals may cause mutations or damage host tissues, which could facilitate viral infections or cause virus escape [6,11,12,13]. For instance, nitric oxide (•NO) may have different effects on host cells after infection with different viruses. In the case of herpes simplex virus (HSV) or Epstein-Barr virus [14,15], •NO has antiviral effects, while for other viruses such as human immunodeficiency virus (HIV) or dengue virus [16,17], •NO can be detrimental to host cells. Similarly, mitochondrial ROS (mtROS) has been shown to have antiviral activity against HIV-1 and influenza A virus, but can be harmful to host cells infected with hepatitis C virus (HCV) [18].

Free radicals can harm cell membranes through various means, such as lipid peroxidation and protein denaturation, leading to tissue damage and cell death. Cell death is an evolutionarily conserved process necessary for maintaining proper function and morphology of healthy tissues. In 2012, Stockwell’s group proposed a new form of cell death called ferroptosis, which differs from traditional forms such as apoptosis, autophagy, and necrosis [19]. The onset of ferroptosis is believed to depend on the iron-dependent accumulation of lipid peroxides. Some research suggests that viruses can interfere with iron metabolism and that iron overload may promote ferroptosis [20,21].

Now, suppressing ferroptosis is widely recognized as a crucial mechanism for inhibiting tumor growth. Extensive research has shown that ferroptosis plays a key role in causing pancreatic cancer, breast cancer, and other forms of tumor cell death. Several studies observed that once combined with the ferroptosis inducer erastin, chemotherapeutic drugs (like cisplatin, doxorubicin/Adriamycin, and temozolomide) gained a remarkable synergistic effect on their anti-tumour activity [22,23,24,25]. Moreover, ferroptosis has a complex relationship with diseases like ischemia-reperfusion injury, degenerative diseases, and acute kidney injury [26]. While few studies have focused on viral infections and ferroptosis as previously stated in introduction, ferroptosis as a programmed cell death, has complex interactions with viruses that still require extensive research. Recent findings, from SARS-CoV-2, HCV, and HIV-1, suggest a strong link between viruses and ferroptosis. Viruses can trigger ferroptosis by disrupting the iron metabolism, antioxidant system, and immune system of host cells through various pathways [20]. Ferroptosis can provide a favorable environment for viruses to survive, replicate, and evade the host immune system [20,21,26]. Therefore, targeting ferroptosis could be a promising approach for antiviral treatment.

In this review, we focus on the impact of ferroptosis on the host immune system during viral infections. We also discuss the potential applications of ferroptosis inhibitors and inducers as a means of antiviral treatment. It is our hope that this review will provide insights that can be used to develop new strategies for designing effective antiviral drugs.

## 2. Free Radical, Iron Metabolism and Viral Infections

### 2.1. Free Radical

**Figure 1:**
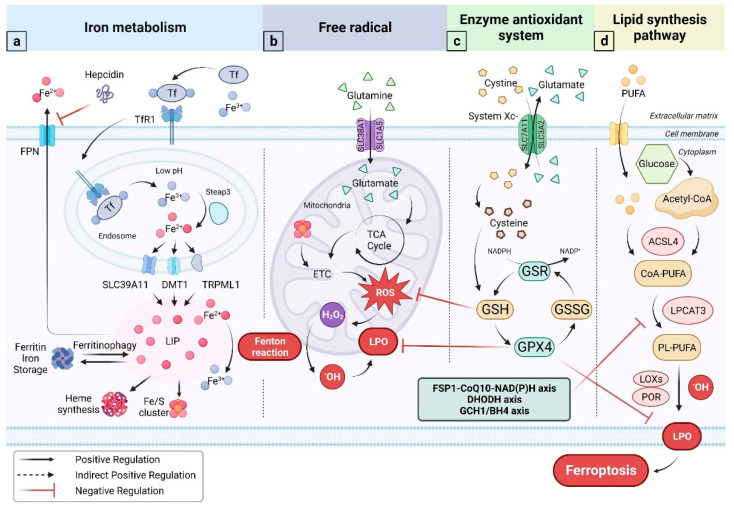
Mechanisms associated with ferroptosis. (**a**) In its Fe^3+^ state, iron loads onto Tf and gets transported through blood to sites with high iron demands. Hepcidin, a major regulator of iron metabolism, binds to FPN and promotes FPN internalization and degradation, suppressing iron efflux. In endosomes with low pH, Fe^3+^ is released and subsequently reduced to Fe^2+^ by Steap3. Fe^2+^ is exported from endosome to cytoplasm through different channels to form LIP and participate in multiple life processes. Some Fe^2+^ are oxidized by going through the Fenton reaction; (**b**) Glutamine enters the cell and participates in the TCA cycle as glutamate in mitochondria. In ECT, excess ROS may be produced. Fe-S clusters can affect the ETC. H_2_O_2_, as a part of the ROS, participates in the Fenton reaction, generating •OH that attacks mitochondrial membrane lipids, and produces LPO; (**c**) cystine, the oxidized form of cysteine, enters the cytoplasm through the System Xc-. Cysteine takes part in the synthesis of GSH, which acts as a reducing substrate and participates in the reaction of GPX4 clear LPO; (**d**) PUFA and acetyl-CoA are converted into CoA-PUFA by the ACSL4 enzyme, which is further transformed into PL-PUFA through the action of LPCAT3. PL-PUFA and •OH produce LPO by LOXs and POR enzymes. LPO accumulation induces ferroptosis. Three GPX4-independent systems for suppressing ferroptosis are listed in the green box. Abbreviations: Tf, Transferrin; TfR1, Transferrin receptor protein 1; FPN, Ferroportin; LIP, Labile iron pool; ETC, Electron transfer chain; ROS, Reactive oxygen species; LPO, Lipid hydroperoxide; System Xc-, Sodium-independent, anionic amino acid transport system; GHS, Glutathione; GPX4, Glutathione peroxidase 4; GSR, Glutathione reductase; GSSG, Glutathione (Oxidized); FSP1, Ferroptosis suppressor protein 1; DHODH, Dihydroorotate dehydrogenase (quinone); CoQ10, Coenzyme Q10; NADPH, Reduced nicotinamide adenine dinucleotide phosphate; Acyl-CoA synthetase long-chain family member; LPCAT3, Lysophosphatidylcholine acyltransferase 3; PL, Phospholipid; PUFA, Polyunsaturated fatty acid; LOXs, lipoxygenases; POR, Cytochrome p450 oxidoreductase.

Under physiological conditions, cells utilize oxidative phosphorylation to transfer electrons from reducing coenzymes to oxygen (Figure 1). This process occurs in the plasma membrane of prokaryotic cells and the inner mitochondrial membrane of eukaryotic cells. However, during electron transport, there is a small probability of electron leakage, which can result in the production of free radicals with high oxidative activity. These free radicals include ROS, lipid peroxides (LPO), and RNS in living organisms. Among these, the most active one is hydroxyl radical (•OH) [27]. As a strong oxidant, •OH attacks cellular phospholipids, causing a chain reaction of lipid peroxidation that damages biofilms and can ultimately trigger ferroptosis (Figure 1) [27].

Viral infections in vivo induce the production of free radicals, which can have both beneficial and detrimental effects on the host. The excess free radicals can cause oxidative stress and damage to cellular and tissue structures, leading to various diseases, such as cardiovascular diseases, liver diseases, kidney diseases, and cancer [12]. In addition, free radicals can interfere with the membrane transport system and mitochondrial respiratory chain resulting in cell injury or even death [28]. The production of free radicals during viral infections is mainly mediated by the NADPH oxidase family (NOX/DUOX) and •NO synthase [29]. Furthermore, several viral infections, including hepatitis B virus (HBV) and HIV, are involved in modulating Ca^2+^ concentrations, which activates NOX5, DUOX1, and DUOX2. Recently, it has been found that respiratory syncytial viral infection can impair mitochondrial respiration, and lead to elevated mitochondrial ROS and loss of mitochondrial membrane potential [13,18].

In recent years, more studies focused on the relationship between viral infections and free radicals from the perspective of ferroptosis. For example, HCV infection can cause iron overload, leading to oxidative stress and ferroptosis [21]. Similarly, SARS-CoV-2 infection has been shown to induce ferroptosis in lung cells, potentially contributing to the severe lung injury seen in COVID-19 patients [30]. Other viruses, such as Newcastle disease virus and Japanese encephalitis (JE) virus, have also been linked to ferroptosis [20,31]. These findings suggest that targeting ferroptosis may have therapeutic potential in the treatment of viral infections.

### 2.2. Iron Metabolism

Iron is the most abundant essential trace element in the human body representing approximately 3–5 g of the total amount [32]. Ferrous (Fe^2+^) and ferric (Fe^3+^) ions are redox-competent and indispensable catalytic centers or cofactors for many enzymes in the body [33]. Additionally, iron ions participate in many vital physical activities, including mitochondrial energy production, immune function, and transcription and replication of genetic materials [34]. In short, iron plays an essential role in maintaining the body’s overall health (Figure 1).

The majority of iron in the body, approximately 90–95%, is obtained through a process known as ‘iron recycling’. This process involves macrophages phagocytosing damaged and senescent red blood cells. The remaining 5–10% of iron is mainly derived from dietary sources and absorbed in the upper part of the small intestine. Once absorbed, iron ions bind to transferrin (Tf) in the plasma, forming the complex transferred bound iron (TBI) (Figure 1). This complex is then transported throughout the body via the bloodstream. Upon reaching the target cell, TBI specifically recognizes and binds to transferrin-receptor (TfR1). Tf-TfR1 then facilitates the invagination of the cell membrane to form the endosome [33]. In a weakly acidic intracellular environment, iron ions are released and participate in various cellular reactions. A small fraction of free or loosely bound Fe^2+^, referred to as the labile iron pool (LIP), can undergo the Fenton reaction intracellularly, resulting in the production of •OH [33]. The maintenance of iron homeostasis is very complex and delicate, involving several links and proteins (or genes), of which hepcidin-FPN is the core of the mechanism regulating iron homeostasis in the body [20,33,35]. Perplexing relationships exist between disrupted iron homeostasis and various complex diseases, such as degenerative diseases, metabolic syndrome, and tumors. In recent years, studies have continued to demonstrate the strong connection between these diseases and ferroptosis [19,36].

The study of ferroptosis has exploded over the past decade. Researchers have found a connection between viral infections and ferroptosis: it was surprisingly discovered that certain viruses specifically hijack iron-rich cells in order to replication. To amass intracellular iron, some viruses alter several mechanisms of iron metabolism. Excess intracellular iron increases the chances of the Fenton reaction, ultimately leading to ferroptosis.

## 3. Ferroptosis

Ferroptosis leads to notable alterations in mitochondria, which can be observed under electron microscopy. These changes include a reduction in size, darkening in color, increased membrane density, absence or reduction in inner membrane cristae, and fragmentation and wrinkling of outer membranes [22]. The cellular components of ferroptosis include the iron metabolism disorder (excess intracellular iron), imbalances in the oxidation-reduction system (depletion of GSH, lower GPX4 activity), increased ROS, and accumulation of lipid peroxides [19,20,26,37,38]. Among the organelles, mitochondria are not only iron-rich (the oxidative respiratory chain depends on iron ions) but also the main places for ROS production. In addition, mitochondria are rich in membrane structures that provide lipid precursors for ferroptosis [39]. Therefore, it is believed that mitochondria are the essential site where ferroptosis occurs.

Numerous metabolic signaling pathways and biological functions regulate ferroptosis [40]. As noticed below, there are three phases to the development of ferroptosis (Figure 1) [26].

The initial stage is the accumulation of intracellular iron and inhibition of the antioxidant system. Expansion of the LIP increases the probability of the Fenton reaction [41], which triggers ferroptosis [42]. There are two basic approaches to enhancing the LIP: either increasing the cellular uptake of iron or inhibiting iron export, such as ferritin autophagy, degradation of iron-containing organelles (mitochondria, lysosomes), increased expression of TfR1 and hepcidin [43,44,45,46]. Regardless of free radicals, the Fenton reaction, or the chain reaction of lipid peroxidation the oxidation reaction causes damage to the body. The body has created a series of antioxidant defense mechanisms to combat the negative consequences of oxidation. The antioxidant system falls into two types [27]. One is the non-enzymatic antioxidant system, which includes melatonin, zinc, selenium (Se), vitamin C, and vitamin E. The other is the enzymatic antioxidant system, which consists of superoxide dismutase (SOD), catalase (CAT), and glutathione peroxidases (GPXs). Superoxide anion (O_2_^•−^), the source of free radicals, is catalyzed by SOD into the comparatively stable molecule H_2_O_2_. CAT catalyzes the reduction in H_2_O_2_ to water and oxygen, thereby inhibiting the oxidation of H_2_O_2_ to •OH. In addition, GSH and Trx(SH)_2_ also can reduce H_2_O_2_. GPXs belong to a family of phylogenetically related enzymes, including GPX1–GPX8, whose key role is to maintain the homeostasis of H_2_O_2_ and function as an antioxidant [47]. To develop GPXs antioxidant activity, GPXs usually use GSH as a reducing substrate (Figure 1). GSH is a tripeptide containing a sulfhydryl group with a component named cysteine (Cys). Cys is transported by a cystine/glutamate antiporter system (System Xc-), whose active core is SLC7A11. Specifically, GPX4, with selenium as the active center, can restore toxic LPO to non-toxic lipid alcohols and is particularly prominent in protecting biological membrane structure and function from oxidative damage [48]. Because of this, GPX4 is regarded as a crucial ferroptosis inhibitor and is also referred to as a membrane lipid repair agent [26]. It is worth considering whether other selenium-containing GPXs also act as an antidote to reduce LPO, in addition to GPX4. Do they exhibit a similar antidotal effect or at least assist in the function of GPX4? In addition to the System Xc-/GSH/GPX4 axis, a few other systems, including the recently identified FSP1-CoQ10-NAD(P)H axis, DHODH axis, and GCH1/BH4 axis, also inhibit ferroptosis [49,50,51,52,53]. The chance of ferroptosis greatly increases when LIP rises and the antioxidant system is inhibited. In this scenario, ferroptosis can be likened to a barrel of dynamite with an ignited fuse.

The accumulation of LPO is the intermediary stage leading to ferroptosis is shown in Figure 1. Two essential enzymes that promote ferroptosis are acylcoenzyme A (CoA) synthetase long-chain family member 4 (ACSL4) and lysophosphatidylcholine acyltransferase 3 (LPCAT3). Inactivation of ACSL4 is a crucial mechanism for the suppression of ferroptosis. Free polyunsaturated fatty acids (PUFAs) in lipid droplets are first bound to Acetyl-CoA by the activity of ACSl4. Subsequently, they are merged into specific phospholipids (PLs) by the effect of LPCAT3 to form PUFA-PLs [54]. PUFA-PLs are the lipid precursor for ferroptosis. Interestingly, not all PUFA-PLs can be lipid precursors for ferroptosis. Lipidomic studies suggest that phosphatidylethanolamines (PEs) are more susceptible to ferroptosis than other PLs [55]. The reason is that the acyl tails on the PEs headgroup (arachidonic acid (AA)- or adrenic acid (AdA)-containing species in phosphatidylethanolamine) are the preferred oxidation substrates and critical to the development of ferroptosis [26,56]. PUFA-PLs and ˙OH are catalyzed by two iron-containing enzymes, named lipoxygenases and cytochrome P450 oxidoreductase, which lead to an oxidation reaction to produce PUFA-PL-OOH, also known as LPO [26]. LPO triggers ferroptosis.

The ultimate phase of ferroptosis involves the accumulation of LPO, cell membrane rupture, content leakage, and finally cell death [57]. However, there are still many unanswered questions regarding this phase. Caspase-3 is the apoptosis effector. Effectors of necroptosis and pyroptosis are mixed-lineage kinase domain-like pseudokinase and gasdermin D, respectively. The end effectors of ferroptosis may be proteins, such as cytochrome p450 oxidoreductase (POR) and cathepsin B, which do not appear to be specific performers of ferroptosis; however [58], they also could be toxic lipids, though there is limited evidence to demonstrate that concentrations of lipotoxic products are high enough to induce ferroptosis [57]. Therefore, further research on the end effectors of ferroptosis is required.

## 4. Ferroptosis and Viral Infections

**Figure 2:**
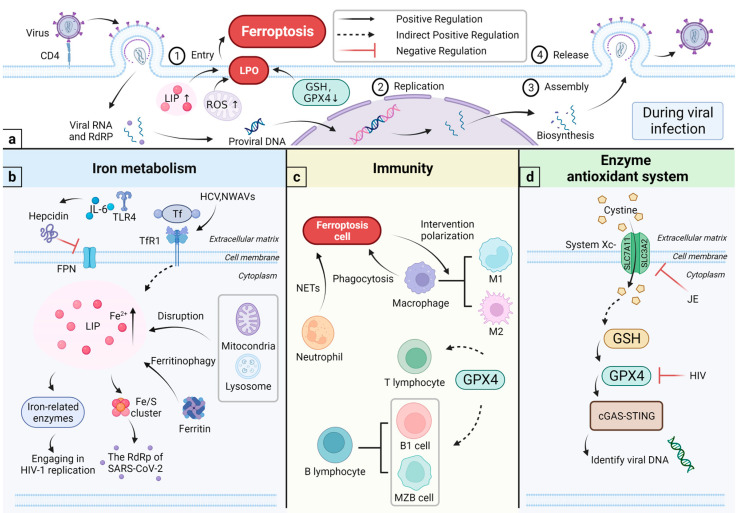
Viral infections and ferroptosis. (**a**) The HIV-1 virus is an example to demonstrate the viral life cycle. The typical viral life cycle can be summarized in four stages: entry into the host cell, viral replication, viral assembly, and release. During viral infections, cells trigger ferroptosis in several ways, for example, by expanding LIP, producing excess ROS, decreasing GSH content, and down-regulating GPX4 activity; (**b**) LIP expansion comes from two aspects: limiting iron ion efflux and increasing intracellular free iron levels. IL-6 and TLR4 pathways increase Hepcidin synthesis and restrict iron ion efflux. Viruses, such as HCV and NWAVs, specifically recognize TfR1 and increase cellular iron uptake. Additional processes, including degradation of iron-rich organelles, ferritin autophagy and other pathways, can also increase LIP levels. Some viruses use iron to assist in their replication: HIV-1 uses an iron-containing ATP-binding enzyme ATPase ABCE1 to assemble the capsid, and SARS-CoV-2 uses Fe-S clusters to make up the protein; (**c**) in concern of immunity, macrophages specifically recognize and engulf ferroptosis cells. Conversely, ferroptotic cells can influence the polarization of macrophages. Neutrophils form NETs, which induce ferroptosis and thus fight pathogens. GPX4 can contribute to the development of lymphocytes. GPX4 helps T cells to fight against some pathogens, but, during GPX4 deficiency, it instead induces ferroptosis in T cells. B1 and MZB cells need GPX4 to maintain and perform immune functions; (**d**) GPX4 is essential for STING activation. The cGAS-STING pathway recognizes viral cell DNA. HIV reduces the intracellular GSH level and or affects GPX4 activities. JE inhibits the expression of systemic Xc-. Abbreviations: NETs, neutrophil extracellular traps; JE, Japanese encephalitis virus.

Viruses can create an environment that is conducive to their multiplication by disrupting cellular energy metabolism and the antioxidant system. Additionally, viruses can subvert immune defense through the induction of ferroptosis, exacerbating diseases (Figure 2).

### 4.1. Relationship among Viral Infections, Iron Metabolism, and Immunity

Iron and its derivatives, such as haemoglobin and Fe-S clusters, are essential for many types of enzymes and are irreplaceable in their centrality in the main processes of cellular physiology. Simultaneously, iron has significance for viruses [21].

#### 4.1.1. Viral Infections and Iron Metabolism

The ultimate purpose of viral infections is virus multiplication. The life cycle of all viruses can be summarized in four stages: entry into the host cell, viral replication, viral assembly, and release, with the specific aspects of detail varying somewhat depending on the virus species (Figure 2) [59]. Iron is essential for viral replication. To replicate, some viruses, such as HCV, gastroenteritis virus, New World hemorrhagic fever virus, and New World sandy viruses, specifically recognize and bind to cells with TfR1, to create an ‘ideal hotbed’ rich in iron [60,61,62,63]. Furthermore, several viruses, including SARS-CoV-2, HIV, and coxsackievirus B3, can travel through various pathways (e.g., hepcidin, DMT1, HFE) to affect iron metabolism and “help” the host cell to absorb iron [64,65,66]. The role of iron has been identified at several stages during HIV-1 replication, as listed below: (1) dNTPs production during the reverse transcription stage, where the iron-dependent ribonucleotide reductase is needed [67]; (2) transcription initiation of the integrated viral genome, where the iron-activated inhibitor kappa B kinase is needed [68]; (3) initiation and prolongation of the gene transcription, where a couple of iron-dependent enzymes (T1-CDK9 and CDK2) are needed [69,70]; (4) expression of the viral structural genes, where an iron-dependent enzyme named deoxy hydroxylase is needed [71]. Moreover, assembling the capsid of the HIV-1 depends on the ATPase ABCE1, which is the only known iron-containing ATP-binding enzyme [72]. Related studies have shown that the Fe-S clusters is a crucial component of certain viral proteins, such as the RNA-dependent RNA polymerase found in SARS-CoV-2 (Figure 2) [73,74].

Ferroptosis is an iron-dependent accumulation of lipid ROS. Intracellular iron overload is important for its initiation. Therefore, cells exhibiting elevated intracellular iron due to viral infections are more vulnerable to ferroptosis. Ferroptosis prevents viral replication and also inhibits the spread. In a HCV study, fatty acid desaturase 2 (FADS2) was found to induce ferroptosis, and RNAi-directed FADS2 knockdown enhanced HCV replication [75,76]. It demonstrates that ferroptosis can inhibit HCV replication. In conclusion, the relationship between ferroptosis and viral replication is complex. Further research data is needed to elucidate the relationship between them.

#### 4.1.2. Iron and Immunity

Iron metabolism tightly links to the immune system (Figure 2). Viruses can interfere with iron metabolism and induce ferroptosis. At the same time, viruses disturb the innate immunity and promote inflammation, thereby exacerbating the disease. Innate immunity includes physiological protective barriers, immunologically active substances, and immune cells. Hepcidin, the regulator of iron metabolism, can degrade ferroportin (FPN) in macrophages and intestinal epithelial cells and inhibits iron efflux [77]. In short, it plays a negative regulatory role in iron homeostasis. Under stimuli such as inflammation, upregulation of hepcidin expression can induce ferroptosis. Moreover, being an acute-phase protein, its synthetics are affected by the inflammatory factors IL-6 and Toll-like receptor-4 [78,79]. Therefore, in some chronic diseases, elevated levels of hepcidin caused by inflammation are thought to be responsible for the accumulation of iron in macrophages and the inhibition of erythropoiesis [80,81].

Innate immune cells, including macrophages and granulocytes, are essential in the body’s defense against and clearance of pathogens. Macrophages exhibit a potent phagocytic capacity. They can engulf and dispose of metabolic waste, cellular debris and pathogens in different cellular forms. At the outer surface of the plasma membrane, cells undergoing ferroptosis express oxygenated phosphatidylethanolamine, which is recognized and cleared by macrophages via TLR2 [82]. Furthermore, macrophages can activate the adaptive immunity through, for instance, lymphocytes. In iron metabolism, macrophages are involved in iron “recirculation” and maintain iron homeostasis in the body. Certain viruses can interfere with iron metabolism through macrophages, causing iron overload in cells and leading to ferroptosis, which limits the immune activity of macrophages [21]. Ferroptosis also affects macrophage polarization (Figure 2) [83]. Macrophages are polarized into two types, M1 and M2, in response to different inducing factors [84]. The imbalance between M1 and M2 can cause disease and inflammation [85]. In concern of resisting ferroptosis, M1-type macrophages can inhibit the activity of ALOX2, by the same token, express higher levels of the enzyme inducible nitric oxide synthase (iNOS) [57]. Neutrophils are the first immune cells for which the inflammatory site is recruited. Reports have shown that neutrophils are involved in the maintenance of inflammation caused by ironophilic tissue damage [86]. In addition, it is thought that neutrophils create neutrophil extracellular traps (NETs), to fight infections by inducing ferroptosis [87].

In HCV studies, viruses can disrupt iron metabolism and cause iron overload in several ways, including (1) the virus interferes with iron metabolism [60,88]; (2) the virus affects genes for iron metabolism in host cells, causing hereditary diseases such as haemochromatosis [89,90]; and (3) the virus inhibits hepcidin production [91]. Increased intracellular iron concentrations in HCV infections facilitate HCV replication, thereby exacerbating oxidative stress and promoting chronic inflammation to aggravate the disease. Simultaneously, it increases the probability of ferroptosis [92]. Based on the above findings, iron can act as a bridge to connect viral infections, ferroptosis and immunity.

### 4.2. Relationship among Viral Infections, Enzyme Antioxidant System, and Immunity

Viruses can trigger the accumulation of free radicals through various mechanisms. When oxidative stress surpasses the reducing capacity in the organism, it leads to increased inflammatory infiltration of neutrophils and secretion of proteases [93]. This results in the massive production of oxidative intermediates that eventually cause tissue damage.

#### 4.2.1. Viral Infections and Enzyme Antioxidant System

The relationship between the System Xc-/GSH/GPX4 axis and viral infections is complex. In the enzymatic antioxidant system, Cys participates and plays a rate-limiting role in the synthesis of GSH [94]. Prior studies noted that Cys is involved in viral protein synthesis, some of which depend on the transport of system Xc- [95]. Another finding in some viral infections is that system Xc- is intact but the antioxidant system is missing. JE can promote neuronal damage by enhancing the activity of system Xc- [95].

#### 4.2.2. Enzyme Antioxidant System and the Immunity

A critical component of the enzyme antioxidant system is the System Xc-/GSH/GPX4 axis, which is powerful in reducing oxidative molecules and has a role in the immune defence system (Figure 2) [96].

Adaptive immunity protects against complex and diverse pathogens in the body. GSH and GPX4 contribute to the development of lymphocytes (Figure 2) [96]. Studies show that GPX4 protects T cells from acute lymphocytic choriomeningitis virus and *Leishmania* major parasite infections [97]. Under non-inflammatory conditions, GPX4 is an essential enzyme for the presence of T cells [97]. Low levels of GPX4 affect the physiological activity of T cells and promotes ferroptosis [98]. Relevant studies prove that the effects of GPX4 deficiency can be remedied by VE supplementation [97,99]. VE, as part of the non-enzymatic antioxidant system, is considered an inhibitor of ferroptosis because of its antioxidant activity [97]. Additionally, GPX4 is necessary for STING activation (Figure 2) [100]. The cGAS-STING pathway recognizes viral DNA, among others [101]. Subsequently, it initiates the innate immunity and can modulate the following adaptive immunity [100]. One study, where HSV-1 was used as a model, demonstrated how the virus disrupts the innate immune response by regulating GPX4 and lipid peroxidation and enabling the immunological escape mechanism (Figure 2). In the adaptive immunity, B lymphocytes have been found to be present in peritoneal and pleural cavities (B1 cells), enriched in the marginal zone of the spleen (marginal zone B [MZB] cells). B1 and MZB cells require GPX4 for maintenance, development, and antibody response. Both cells express higher levels of fatty acid transporter protein CD36 so that they have greater uptake of fatty acids and are more sensitive to ferroptosis [102].

As mentioned above, viruses affect the body by disrupting multiple systems including iron metabolism, antioxidant system, and immune defence system. Disruption of the iron metabolism results in iron deficiency anaemia, which leads to a slow recovery and poor prognosis. Moreover, disruption of the immune system facilitates infection by secondary pathogens, which not only exacerbates the disease but also makes treatment difficult.

## 5. Intervention of Viral Infections through Ferroptosis

### 5.1. Ferroptosis Inducers and Viral Infections

Ferroptosis is regulated by a board of cellular metabolic pathways, including redox homeostasis, iron homeostasis, energy metabolism (amino acids, lipids, and so on), and multiple signalling pathways associated with the disease. As a regulatory cell death modality, triggering ferroptosis would be advantageous to disease treatment by clearing cells with obvious or potential problems, such as cancer cells, inflammatory cells, or activated fibroblasts [26]. Stockwell argues that ferroptosis inducers may be beneficial in the context of cancers and infectious diseases [26].

So far, there are four categories of ferroptosis inducers: (1) type I FIN inhibits system Xc-; (2) type II FIN inhibits/degrades/inactivates GPX4; (3) type III FIN depletes reduced coenzyme Q10; and (4) type IV FIN induces lipid peroxidation via peroxide, iron or polyunsaturated fatty acid overload. Current studies found that type I and II FIN have better clinical promise. With different removal targets and methods, the choice of ferroptosis inducers needs to be chosen carefully. The ferroptosis inducers exhibit specificity and do not elicit activation of markers associated with other cell death types during induction. In recent years, ferroptosis inducers have been used to eliminate various types of cancer cells. Studies showed that erastin can induce iron toxicity in gastric cancer cells by limiting the synthesis process of GSH [103]. In addition, it can also trigger ferroptosis in colorectal cancer cells through other pathways [104,105]. Artesunate specifically induced ROS production and activated ferroptosis in ovarian cancer and pancreatic ductal adenocarcinoma cell lines [106,107].

Less research has been conducted on the application of ferroptosis inducers in infectious diseases. HBV can prevent iron overload by secreting miR-222 [108], it activates the NRF2-GPX4 axis through EBNA1 to inhibit ferroptosis and maintain cell survival. Surprisingly, HBV also promotes hepatic fibrosis and nasopharyngeal carcinoma tumourigenesis, eliminating the effects of chemotherapy (resistance to cancer treatment drugs) [109,110]. Sorafenib, a ferroptosis inducer, triggers hepatic stellate cell ferroptosis, while it attenuates liver injury and fibrosis via HIF-1α/SLC7A11 signalling [111]. Merkel cell carcinoma (MCC) is a rare and aggressive form of skin cancer, often found in the elderly population, with a low overall survival rate of about 54% [112,113]. MCC has a high rate of local recurrence and lymph node metastasis. In addition, the disease is difficult and costly to treat. It has been found that nearly 80% of cases are associated with Merkel cell polyomavirus (MCPyV) [113]. Artesunate is a semi-synthetic derivative of artemisinin, which treats malaria and has antiviral activity against many viruses [114]. Furthermore, it is an ferroptosis inducer [115]. It has been demonstrated that artesunate can inhibit the proliferation of MCC cells by triggering ferroptosis in MCC cells when the fine is infected with MCPyV and becomes MCC cells [116].

### 5.2. Ferroptosis Inhibitors and Viral Infections

Just like inducers, ferroptosis inhibitors can be used in tumour immunotherapy and other diseases. Inhibiting ferroptosis lies in the direct blocking of specific lipid peroxidation reactions. Based on the mechanism of action of ferroptosis inhibition, they are divided into three types, as follows.

First, some inhibitors enhance the antioxidant system, particularly those associated with the systemic Xc-/GSH/GPX4 axis. GPX4 acts as a central inhibitor of ferroptosis [117]. Its upregulation directly prevents ferroptosis. The active centre of GPX4 is selenium (Se) [47]. Among the GPXs mimetics currently developed, the selenoorganic compound ebselen (2-phenyl-1,2-benzisoselenazol-3(2H)-one), has broad specificity for organic hydroperoxides as well as substrates of membrane-bound phospholipids and cholesterol hydroperoxides. Oral ebselen has been shown to be well-tolerated and therapeutically effective in several studies [118]. The use of N-acetylcysteine (NAC) and GSH ester derivatives can enhance GSH [119]. Within cells, water-soluble NAC deacetylates in cells to produce Cys. It replenishes Cys in the presence of systemic Xc- suppression. GSH ester derivatives are lipophilic, they can be rapidly hydrolysed and form glutathione by non-specific esterases upon entry into the cell [120,121]. In addition, by increasing GSH, Fer-1 rescues ferroptosis resulting from overactive P53 [122].

The second is to regulate the ACSL4 and lipid substrate related with ferroptosis. ACSL4 is essential for the production of ferroptosis lipid substrates. Ferroptosis inhibitors are found in drugs for the type 2 diabetes treatment. There are three agonists of PPARγ, including troglitazone, rosiglitazone and pioglitazone [123]. They block the activation of PUFA and the phospholipidation process, thereby reducing the production of lipid peroxidation precursors.

The third is to control the amount of intracellular iron. Viral infections can increase intracellular iron concentrations, which aggravates the condition. Therefore, limiting iron to infected cells can inhibit viral replication and prevent certain virus-induced cell enlargement phenomena. The reduction in free iron in the cytoplasm is mainly through iron chelators. Several studies have demonstrated that iron chelators can limit HIV-1 replication in multiple ways. For example, the chelators ICL670 and 311 can inhibit Tat-induced HIV-1 transcription in CEM-T cells by affecting the cell cycle proteins CDK2 and CDK9 [124]. Desferrioxamine blocks the activation of HIV-1 [125,126]. In addition, the drug, in combination with hydroxyurea, can inhibit the production of viral particles. Some large DNA viruses, such as cowpox and HSV-1, possess the genetic information for iron-dependent ribonucleotide reductase [127,128]. The iron chelator 2,2′-bipyridine is used to prevent viral replication.

Ferroptosis is a well-known cause of many organ injuries and degenerative diseases. Therefore, in pharmacology, the use of medications that either induce or inhibit ferroptosis holds great promise for the treatment of many diseases. Examples include the treatment of cancer, ischaemic organ damage, and degenerative diseases associated with widespread lipid peroxidation. In recent years, novel inhibitors and inducers of ferroptosis have been continuously discovered, and the mechanisms are being increasingly studied in depth and comprehensively. Currently, the most studied viruses associated with ferroptosis are viral hepatitis, HIV, and JE [76,95,129,130]. Research on the association between SARS-CoV-2 and ferroptosis is also constantly increasing. However, a targeted exploration of the pathophysiological mechanisms between a particular viral disease and ferroptosis is still necessary. Investigation into the interaction between viruses and ferroptosis will aid in the development of suitable inhibitors or inducers, thereby facilitating drug development and therapeutic regimen design.

## 6. Conclusions and Outlook

The severity of viral infections is determined by two aspects: (1) pathogen virulence, including the viral pathogenicity, the amount of virus present, and the suitable port of virus entry; (2) host susceptibility, which mostly refers to the host immune system. Viruses exploit cellular functions, leading to host inflammation and tissue damage in hosts. Viral infections induce oxidative stress in the host cells, releasing large amounts of free radicals, under the action of which viral replication is enhanced. Meanwhile, excessive free radicals within an organism can cause damage and lead to disease. On the other hand, cells exert antiviral strategies, such as regulated cell death. Ferroptosis is a newly discovered form of regulated cell death and has attracted increasing attention.

To acquire sufficient resources and establish a favourable environment for reproduction, the virus manipulates various cellular processes, including but not limited to glucose metabolism, lipid metabolism, amino acid metabolism, redox homeostasis, and iron metabolism. Excess intracellular iron can trigger the Fenton reaction that generates a potent oxidant known as •OH. The ubiquitous intracellular phospholipid bilayer is an abundant source of lipid precursors. Oxidation of lipid precursors by •OH produces LPO, leading to ferroptosis. In the context of viral infections, ferroptosis can act as a suicidal defense to halt the replication and propagation of viruses. Excess intracellular iron and oxidative stress can disrupt the host immune defense system. The induction of hepcidin expression by inflammatory factors (such as interleukin IL-6) or inflammatory proteins like ferritin is a crucial factor in the occurrence of pathological iron homeostasis. Hepcidin confines iron to macrophages and prevents viruses from using the iron. Abundant iron supports the activities of many key enzymes for viral replication. Iron accumulation not only induces ferroptosis but also exerts its influence in other areas. GPX4 and GSH can influence lymphocyte maintenance, development, and immune response. Apart from the studies on the mechanisms of ferroptosis in three aspects including energy metabolism, control of ROS as well as iron metabolism, there are also many other studies focusing on the relationship between ferroptosis and organelles [49,131,132].

Currently, several clinical drugs have been found to induce ferroptosis in cancer cells. Toyokuni et al. reported that sulfasalazine inhibits Cys2 uptake via system xc-, resulting in ferroptosis in glioma cells [133]. Lapatinib, one kind of tyrosine kinase inhibitor, can trigger ferroptosis in breast cancer cells when used together with siramesine [134]. Selective management of ferroptosis is a well-accepted therapeutic approach in the treatment of diseases such as cancer. Nevertheless, there is still a lack of theoretical justification for its application to viral infections. Undeniably, using ferroptosis as an antiviral treatment option holds great potential. Controlling ferroptosis requires adequate rational interactions between the virus and the ferroptosis. The key to selectively activating or inhibiting ferroptosis in specific tissues and cells, thereby treating the disease. Various technologies optimize drug delivery, biodistribution, and pharmacokinetics to facilitate more precise control of ferroptosis. For instance, certain nanomaterials can assist in drug delivery. Some recent studies have shown that nanomaterials can regulate ferroptosis by affecting ROS and GSH levels and the Fenton reaction, coordinating the loaded drug [135]. Dietary iron supplementation can contribute to drug therapy and prognosis. However, a low-iron diet may maintain the body’s health and enhance immunity function [88]. T follicular helper cells (Tfh) play a critical role in protective immunity, helping B cells produce antibodies against pathogens. The amount of Tfh is regulated by GPX4-controlled cell death [136]. Enhancing GPX4 abundance through selenium supplementation increases antibody responses after vaccination against influenza [136]. However, the benefits of selenium supplementation are limited. These findings imply that diet may be an influential factor in susceptibility to ferroptosis [137]. It is possible that the optimization of diet could exert control ferroptosis in humans and animals.

## Data Availability

Not applicable.
